# Automatic speech analysis for detecting cognitive decline of older adults

**DOI:** 10.3389/fpubh.2024.1417966

**Published:** 2024-08-08

**Authors:** Lihe Huang, Hao Yang, Yiran Che, Jingjing Yang

**Affiliations:** ^1^School of Foreign Studies, Tongji University, Shanghai, China; ^2^Research Center for Ageing, Language and Care, Tongji University, Shanghai, China; ^3^School of Aerospace Engineering and Applied Mechanics, Tongji University, Shanghai, China

**Keywords:** cognitive decline, natural language processing, machine learning, automatic speech recognition, language aging

## Abstract

**Background:**

Speech analysis has been expected to help as a screening tool for early detection of Alzheimer’s disease (AD) and mild-cognitively impairment (MCI). Acoustic features and linguistic features are usually used in speech analysis. However, no studies have yet determined which type of features provides better screening effectiveness, especially in the large aging population of China.

**Objective:**

Firstly, to compare the screening effectiveness of acoustic features, linguistic features, and their combination using the same dataset. Secondly, to develop Chinese automated diagnosis model using self-collected natural discourse data obtained from native Chinese speakers.

**Methods:**

A total of 92 participants from communities in Shanghai, completed MoCA-B and a picture description task based on the Cookie Theft under the guidance of trained operators, and were divided into three groups including AD, MCI, and heathy control (HC) based on their MoCA-B score. Acoustic features (Pitches, Jitter, Shimmer, MFCCs, Formants) and linguistic features (part-of-speech, type-token ratio, information words, information units) are extracted. The machine algorithms used in this study included logistic regression, random forest (RF), support vector machines (SVM), Gaussian Naive Bayesian (GNB), and k-Nearest neighbor (kNN). The validation accuracies of the same ML model using acoustic features, linguistic features, and their combination were compared.

**Results:**

The accuracy with linguistic features is generally higher than acoustic features in training. The highest accuracy to differentiate HC and AD is 80.77% achieved by SVM, based on all the features extracted from the speech data, while the highest accuracy to differentiate HC and AD or MCI is 80.43% achieved by RF, based only on linguistic features.

**Conclusion:**

Our results suggest the utility and validity of linguistic features in the automated diagnosis of cognitive impairment, and validated the applicability of automated diagnosis for Chinese language data.

## 1 Introduction

The issue of diagnosing Alzheimer’s disease has garnered considerable attention from scholars. However, given the challenges associated with traditional biomarker screening, some researchers have attempted to use machine learning techniques to analyze the speech of older adults as a novel automated screening tool. The features used in machine learning can be concluded into acoustic set and linguistic set.

### 1.1 AD diagnosis

Alzheimer’s disease is a neurodegenerative condition characterized by the progressive decline of cognitive function, such as executive function, inference ability, memory, etc., making it a prominent cause of mortality among the older population (1). Despite considerable research endeavors dedicated to developing disease-modifying treatments for AD, a definitive cure remains elusive. Consequently, the importance of early and efficient prediction of AD cannot be overstated. Biomarkers such as β-amyloid (Aβ), phosphorylated tau protein (p-tau), magnetic resonance imaging (MRI) and positron emission tomography (PET) are utilized to aid in the diagnosis of AD. However, these biomarkers are hindered by limitations such as invasiveness, high cost, and time consumption. Consequently, scholars are actively investigating more efficient and convenient biomarkers.

### 1.2 Automated diagnosis based on acoustic features

Several endeavors have been undertaken to leverage language and speech data gathered from everyday life through computational speech processing techniques for automated diagnosis, prognosis, or progression modeling (2). A prevalent approach in recent research involves utilizing speech data obtained from various language tasks and employing speech processing techniques to extract diverse types of features for modeling (2–4). It is gathering prominence due to their numerous prospective benefits, such as non-invasiveness, cost-effectiveness, and ease of accessibility.

Machine Learning has demonstrated significant effectiveness in language modeling, prompting some research studies to propose its utilization for detecting AD due to its exceptional performance in binary AD vs. control comparisons (5, 6). Gonzalez-Moreira et al. (7) conducted a study where prosodic features were measured in individuals with mild dementia and healthy controls, utilizing automatic prosodic analysis during a reading task. The study achieved a classification accuracy of 85%, indicating a noteworthy discriminatory capacity between the two groups. Roshanzamir et al. (8) achieved an accuracy rate of 88.08% by employing BERT as an encoder in conjunction with a logistic regression classifier to differentiate between AD patients and controls, utilizing English data from the Pitt corpus.

The features employed in automated diagnosis research are mainly acoustic-dependent features. Acoustic-dependent features encompass characteristics that do not necessitate comprehension of content, including prosodic features such as pause rate, articulation rate, and spectral features like formant trajectories and Mel Frequency Cepstral Coefficients, as well as vocal quality indicators like jitter and shimmer (as listed in Table 1). Satt et al. (27) recorded participants during various cognitive tasks and meticulously engineered acoustic features tailored to each task. Their study achieved an equal error rate of 87% in a binary classification distinguishing between AD and HC groups. Balagopalan et al. (21) modeled the acoustic part of speech using acoustic parameters of frequency and spectral domain by patients’ verbal descriptions of the Cookie Theft picture. In their study, Support Vector Machine (SVM) had the highest performance with an accuracy of 81.5%. Rohanian et al. (28) modeled the acoustic part of speech using COVAREP, an open-access repository of acoustic assessment algorithms. An ML architecture with Bi-long short-term memory (Bi-LSTM) was trained and evaluated on the linguistic and acoustic feature sets, achieving an accuracy of 79.2% for the joint combination of acoustic and linguistic feature sets. Balagopalan et al. (5) employed MFCCs, fundamental frequency, and zero-crossing rate-related statistics as acoustic parameters, together with lexical-semantic features, achieving an accuracy of 81.3% with SVM model.

**Table 1:** Acoustic feature taxonomy.

Subcategory	Feature type	Feature name	References
Prosodic features	Temporal	Pause rate (PR)	(9, 10)
		Hesitation rate	(11, 12)
		Speech rate (SR)	(11)
		Articulation rate (AR)	(11–13)
		Speech tempo	(11, 12)
	Fundamental frequency	F0 and trajectory	(14, 15)
Spectral features	Formant trajectories	F1, F2, F3	(16–20)
	Mel frequency cepstral coefficients	MFCCs	(21)
Vocal quality	Jitter, Shimmer, harmonic-to-noise ratio	Jitt, shimm, HNR	(22–24)
ASR-related	Filled pauses, repetition, dysfluencies, hesitations, fractal dimension, entropy.	FP, rep, dys, hes, FD, entr	(25)
	Dialog features (i.e., turn-taking)	TT: avg. turn length, inter-turn silences	(26)

In addition to the acoustic features utilized in the research articles mentioned before, there are also standard acoustic feature sets such as ComParE (29), GeMAPs (30), and eGeMAPS, which are supported by the openSMILE v2.4.2 toolkit based on the Python languge. The ADReSS Challenge at INTERSPEECH 2020 (31) also suggests other feature sets such as emobase (32) and MRCG functions.

### 1.3 Automated diagnosis based on linguistic features

In addition to the measurement of acoustic features, some scholars believe that linguistic indicators can better reflect changes in patient’s language abilities.

Language impairment represents a prominent manifestation of AD, characterized by challenges in both speech production and comprehension. This symptomatology typically emerges in the early stages of the disease and deteriorates in tandem with disease progression. Moreover, research indicates that individuals with manifest AD exhibit indications of language deficits long before receiving a formal diagnosis (33). This observation proves particularly valuable in identifying mild cognitive impairment (MCI), which serves as the prodromal stage of AD (34).

Based on the disproportionate impairments in language functions at various stages of Alzheimer’s disease, some scholars have proposed using linguistic features for automatic diagnosis.

Linguistic features pertain to aspects related to the meaning, grammar, or logic of speech. These features encompass diverse linguistic domains and corresponding attributes, including lexical diversity and density at the lexical level, dependency-based parse tree scores at the syntactic level, latent semantic analysis at the semantic level, as well as assessments of coherence, paraphrasing, and filler words at the pragmatic level (2). The linguistic features used for automated diagnosis are listed in Table 2. Sadeghian et al. (44) employed the extracted linguistic features to augment the Mini-Mental State Examination (MMSE) for AD detection resulting in a substantial improvement in accuracy from 70.8% to 94.4%. Guo et al. (45) conducted a lexical-level analysis using N-grams LMs for AD detection and achieved state-of-the-art detection accuracy of 85.4% on the DementiaBank dataset. Balagopalan et al. (5) utilized lexico-syntactic features to model the linguistic aspects of participants’ speech. These features were derived from speech-graph, constituency parsing tree, lexical richness, syntactic, and semantic features based on picture description content.

**Table 2:** Linguistic feature taxonomy.

Subcategory	Feature type	Feature name, abbreviation	References
Lexical features	Bag of words	BoW	(35)
	Linguistic inquiry and word count	LIWC	(36, 37)
	Lexical diversity	Type-token ratio (TTR)	(38, 39)
		Moving average TTR (MATTR)	(38)
		Brunet’s index; Honore’s statistic	(40–42)
		Familiarity; imageability; age-of-acquisition	(38)
	Vocabulary analysis	The total number of utterances; mean length of utterances; functional words; unique words; word count; character length; lexical bigrams	(25)
	Part-of-speech tagging	PoS	(26, 37, 38)
	Lexical density	Idea density (ID)	(43)
Syntactical features	Constituency-based parse tree scores		(38)
	Sentence analysis	The mean length of sentences; T-units; clauses	(38)
		The frequency of occurrence of different grammatical constituents	(38)
		The rate, proportion, and average length of noun phrases, verb phrases, and prepositional phrases	(38)
		Coordinated sentences; subordinated sentences; reduced sentences; number of predicates; average number of predicates; dependency distance; number of dependencies; average dependencies per sentence	(25)
Pragmatics	Coherence	The filler words; phrase repetitions; word revisions; phrase revisions	(26)

### 1.4 Comparison between acoustic and linguistic features

Upon comparing the two types of indicators, it can be observed that the advantages of acoustic features lie in their strong transferability and independence from linguistic content. Minimal effort is required to apply this methodology to another language (46). Algorithms focusing on acoustic features demonstrate universality, allowing researchers from diverse countries to study various languages and speech tasks. Consequently, research centered on acoustic features enjoys broader prevalence and application within academic circles. However, acoustic features are heavily reliant on audio quality. Poor audio quality may adversely affect the detection efficacy.

Compared to acoustic features, studies utilizing linguistic features often achieve higher performance levels due to their richness in information. These indicators involve deeper levels of language proficiency, thus better reflecting changes in patients’ language abilities. However, these features necessitate a more in-depth analysis of discourse content, hence, the generation of spoken content typically requires automatic speech recognition (ASR) before relevant contextual analysis can be conducted. Their robustness is highly dependent on the quality of the ASR system (46). In addition, relevant algorithms must also consider the uniqueness of the language, combing linguistic features of this language (such as English, Chinese etc.) to better analyze syntactic, pragmatic performance of AD patients. Therefore, these algorithms are designed for specific languages. While context-dependent features may have limitations in transferability, preventing direct application to other languages, they still hold significant research value.

Several studies have integrated both acoustic and linguistic features in their research. Fraser et al. (47) investigated speech and corresponding transcriptions from 240 AD patients and 233 healthy controls in the DementiaBank corpus. They extracted a total of 370 features, encompassing aspects such as part-of-speech (POS), syntax, acoustic properties, and other linguistic factors. Their study achieved a best average accuracy of 81.92% for binary classification. Similarly, Lopez-de-Ipiña et al. (22) utilizing Hungarian data, achieved accuracy rates ranging from 72% to 82% in classifying cognitively healthy individuals, those with MCI, and those with AD based on acoustic features, with comparable results obtained using language features. Furthermore, He et al. (33) employed seven speech and linguistic features in a random forest classifier to assess the discriminability of participants from Spanish/Catalan backgrounds with AD, MCI, subjective cognitive decline (SCD), and cognitively healthy controls, obtaining scores exceeding 90% in their evaluations. Kong (48) modeled the linguistic aspects of speech by employing syntactic and semantic features, as well as psycholinguistic characteristics. For acoustic parameters, they utilized the MFCC features. They combined the two modalities using a joint embedding method and built logistic regression classifiers on these feature sets, achieving an accuracy of 70.8%.

### 1.5 Existing research limitations

Considering the somewhat surprising quantity and diversity of studies we encountered, it is reasonable to conclude that this is a highly promising field. However, it is evident that several challenges need to be addressed.

Firstly, the primary limitation lies in the comparison of the diagnosis effectiveness between acoustic features and linguistic features. Although both types of indicators can be used for early screening of AD, there seems to be a lack of comparative studies on which type is more effective. Most existing studies utilize only one type of indicator or combine both types, seemingly assuming that the combination of both indicators yields better diagnosis results. However, no study has yet compared the diagnosis effectiveness of acoustic features, linguistic features, and their combination using the same dataset. A comparative analysis of these three indicator combinations would enhance our understanding of the effectiveness of acoustic and linguistic features. It would help improve the accuracy of machine learning and serve as a reference for scholars in choosing indicators for related research in the future.

Secondly, automated speech analysis has predominantly focused on English speech data. Notably, there has been a dearth of research specifically targeting Chinese language data, with only a sparse representation in the comprehensive systematic analysis conducted by Garcia et al. (2), wherein merely one study (46) was conducted on Chinese and Taiwanese datasets. However, given the burgeoning interest in studying language and aging within the Chinese older population, it is widely acknowledged among scholars that employing machine learning techniques on Chinese speech data holds substantial promise for enhancing AD detection efforts. This recognition is imperative for the global dissemination and implementation of robust screening methodologies for AD.

### 1.6 Objects of this research

Hence, this study endeavors to:

1. Compare the diagnosis effectiveness of acoustic features, linguistic features and their combination using the same dataset.
2. Develop a Chinese language analysis model for older adults with cognitive decline using self-collected natural discourse data from native Chinese speakers.

**Figure 1:**
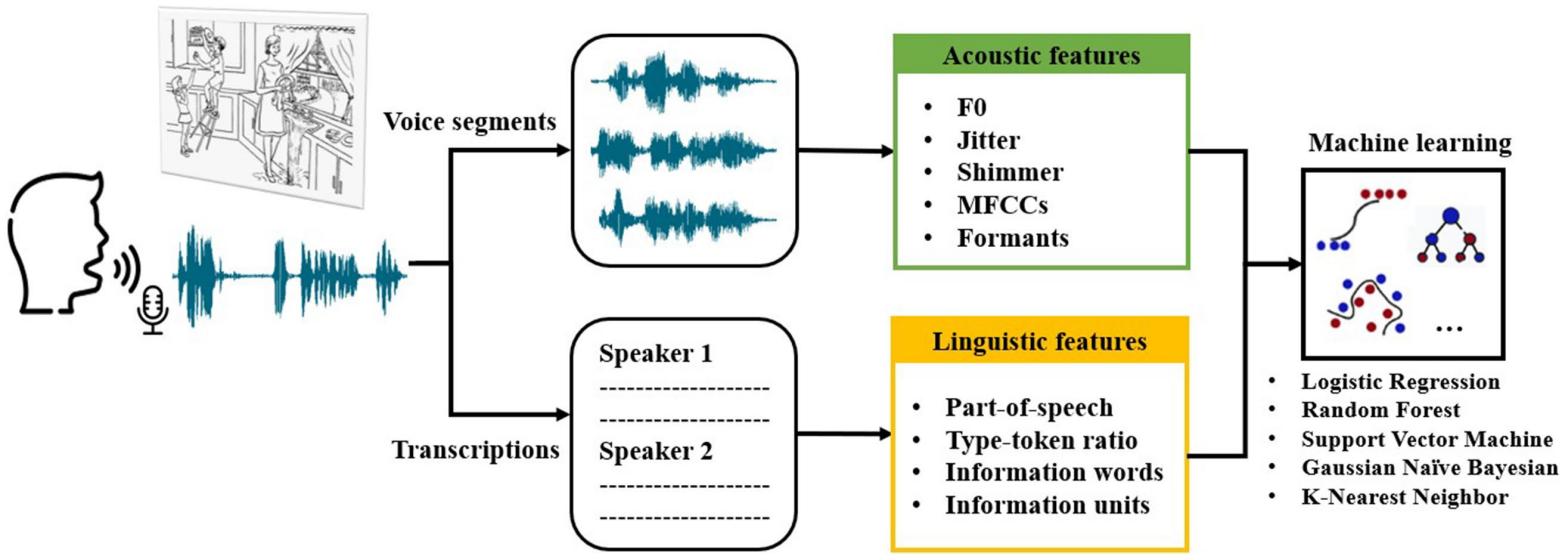
Overview of speech collection and analysis steps as well as machine learning classification in this study.

The study outlined herein follows a structured approach, as depicted in Figure 1. Initially, the voice responses of subjects from Shanghai communities were recorded during a picture description task. Subsequently, voice segments and transcriptions were obtained from the recordings. Following data preprocessing, specific acoustic features were extracted from the voice segments, and linguistic features were extracted from the transcriptions. Finally, the extracted features were employed to train machine learning models for the classification of AD, MCI, and HC groups.

## 2 Materials and methods

### 2.1 Subjects

Subjects were recruited from communities in Shanghai. All participants completed a picture description task based on the Cookie Theft picture from The Boston Diagnostic Aphasia Examination (49) and MoCA-B test under the guidance of trained operators. All subjects were divided into 3 groups including AD, MCI, and HC, based on their MoCA-B score.

Specifically, older adults in AD group fulfilled the following criteria: MoCA-B score <13 and years of education <6, MoCA-B score <15 and years of education range from 7 to 12, MoCA-B score <16 and years of education more than 12; MCI group: MoCA-B score range from 19 to 14 and years of education <6, MoCA-B score range from 22 to 16 and years of education range from 7–12, MoCA-B range from 24 to 17 and years of education more than 12; HC group: MoCA-B score range from 30 to 20 and years of education <6, MoCA-B score range from 30 to 23 and years of education range from 7 to 12, MoCA-B range from 30 to 25 and years of education more than 12. For the consistency in the later acoustic features analysis, we excluded subjects who speak only Shanghainese and preserved only the noise-free Mandarin samples.

Finally, based on the criteria, 92 older adults (40 male and 52 female) ranging in age from 53 to 87 (*M* = 69.82, SD = 8.82) were recruited, including 30 AD patients, 40 MCI patients and 22 HC participants. All older adults were right-handed and native speakers of Chinese. The number subjects in each age group are shown in Table 3. We recorded the speech of each subject using voice recorder Sony ICD-SX2000.

**Table 3:** Basic characteristics of the subjects.

	AD (30)		MCI (40)		HC (22)		*P*-value
	M	SD	M	SD	M	SD	
Age (year)	73.30	9.322	69.90	8.089	64.95	7.319	0.003
Gender (#female)	19	63.33%	23	57.5%	10	45.45%	0.432
Education (year)	8.77	5.036	10.73	4.095	10.82	4.479	0.145
MoCA-B score	10.03	3.200	19.98	2.616	24.73	2.251	0.000

M, mean; SD, standard deviation (number and proportion of females for the gender; more details in Supplementary Appendix 1).

The study was conducted under the approval of the Ethics Committee, Tongji University, under the approval number tjsflrec202306. All participants provided written informed consent to participate in the study. All examinations were conducted in Chinese.

### 2.2 Data collection and analysis

#### 2.2.1 Preprocessing

After collecting speech data from the subjects, researchers used Feishu (50), an online open-source platform for audio processing, to identify the timing of the utterance of each audio file and transcribe the recording. Next, the researchers cut out the subjects’ utterances from the original recording with Parselmouth, a Python interface to the internal Praat code (51) which enables batch analysis of multiple sound files. The voice segments of each subject were joined together as a new audio file and automatic noise reduction in Praat was applied on the concatenated audio file. The transcriptions of the utterances of the subject were extracted and stored in text files.

After the recordings were preprocessed as described above, the audio files and the transcriptions were further used for acoustic and linguistic analysis. All the acoustic and linguistic features we extracted are listed in Table 4. Detailed descriptions of these features can be found in sections 2.2.2 and 2.2.3, respectively.

**Table 4:** Summary of all the acoustic and linguistic features extracted from speech data.

Acoustic features	
F0s	Mean F0s of each utterance
Jitter, Shimmer	Jitter and Shimmer of the joined utterances
MFCCs	Frame-level MFCC1-7 of the joined utterances
Formants	Formants 1–4 of the joined utterances
Linguistic features	
Part-of-speech	Ratio of nouns, verbs, and pronouns, in the total number of words
Type-token ratio	Type-token ratio of words and type-token ratio of characters
Information words	Ratio of information words in the total number of words (Information words include nouns, verbs, adjectives, and numerals)
Information units	Number of people, objects, places, and actions category (specific to the Cookie Theft)

#### 2.2.2 Acoustic features extraction

Recent studies on machine learning approaches based on speech data investigate various acoustic features for classification. For example, Yamada et al. (3) compute the jitter, shimmer, and the variances of MFCC1-12 from the collected speech data. Lopez-de-Ipiña et al. (22) and Gonzalez-Moreira et al. (9) use acoustic features only for training and achieve a considerable accuracy.

By examining the standard acoustic feature sets (9, 29–31, 50) as well as the acoustic features used in several research papers (2, 3, 32, 44, 48, 51) mentioned in section 1.2, we found that the commonly used acoustic features include F0s, jitter, shimmer, MFCCs, and formants. We obtained these acoustic features of our speech data using Parselmouth, and computed their statistics for training ML models.

- Fundamental frequency (F0)

F0 is a measurement of acoustic production based on the rate of vibration of the vocal fold. The F0 of a voice at different times is computed using short-time Fourier transform over a sliding time window. In this study, the F0s were estimated based on the voice recordings of the subjects. For each voice segment of a subject, we extracted the F0s at all the time points of that segment and computed their mean value. After obtaining the mean F0s of all the voice segments, we ranked these mean F0s in ascending order and took the middle seven values for ML classification (median value, three values smaller than and closet to the median value, and three values larger than and closet to the median value). The median F0 value and the three values closest above and below the median could capture the central tendency of the F0 distribution and reflect its variability to some extent, which we believe can be informative for Alzheimer’s detection.

- Jitter and Shimmer

Jitter and shimmer measure the variation of the frequency and amplitude of sound. For each subject, we concatenated all the voice segments into a single audio file and obtained its relative, local jitter and shimmer. The voice segments were concatenated better obtain representative values for each subject. The formula of relative, local jitter and shimmer implemented in Praat are as follow.

$\mathrm{Jitter}=\frac{\frac{1}{N-1}\sum_{i=1}^{N-1}\left|T_{i}-T_{i+1}\right|}{\frac{1}{N}\sum_{i=1}^{N}T_{i}}$ $\mathrm{Shimmer}=\frac{\frac{1}{N-1}\sum_{i=1}^{N-1}\left|A_{i}-A_{i+1}\right|}{\frac{1}{N}\sum_{i=1}^{N}A_{i\text{.}}}$

where *N* is the number of extracted F0 periods, *T_i_* is the length of the *i*^th^ period measure in ms, and *A_i_* is the amplitude of the *i*^th^ period represented on a scale from 0 to 1. The F0 periods are identified using the “To PointProcess” object in Praat, and the corresponding amplitudes are estimated.

- Mel-frequency cepstral coefficients MFCCs

MFCCs describe the distribution of power over different frequencies of a sound signal. Some researches on AD speech recognition use the mean values or other statistics of the first several frame-level MFCCs for training (2, 3, 45, 38). For each subject, we obtained the first 6 frame-level MFCCs of the concatenated speech. The parameters for computing the MFCCs were default values in Praat (window length = 0.015, time step = 0.005). For each of the first 6 MFCCs, we computed the mean values and standard deviations over all the frames for ML classification. Using the mean and standard deviation of the first few frame-level MFCCs is a common approach in speech-based cognitive screening research, as the lower-order MFCCs tend to capture the most essential characteristics of the spectral envelope.

- Formants

Formants are frequency peaks in the spectrum of a sound or signal in general. In acoustic analysis, a formant typically results from a resonance of vocal tract. To extract the formants based on the voice recordings, we first specified the time points of all the pulses using the “To PointProcess” object in Praat, and then extracted the first four formants at these identified pulses. The median value of each formant over the pulses were obtained, resulting in four median formants, which were used for training ML models. The first four formants were chosen since lower-frequency formants are generally more robust and informative for speech analysis.

#### 2.2.3 Linguistic features extraction

There have been a large number of studies showing that cognitive decline is associated with the change of linguistic features of daily speech. For example, Guinn et al. (42) conducted discriminative analysis of conversational speech involving individuals suffering from Alzheimer’s disease and showed that metrics such as part-of-speech rate, type-token ratio, and Brunet’s index, contrast between residents diagnosed with Alzheimer’s disease and their healthy caregivers. Vincze et al. (52) suggested that morphological features including number of words and rate of nouns, verbs, adjectives, help distinguish MCI patients from healthy controls. Ahmed et al. (53) found that measures of semantic and lexical content and syntactic complexity best capture the global progression of linguistic impairment through the successive clinical stages of disease. Lira et al. (54) reported that decreased performance in quantity and content of discourse is evident in patients with AD from the mildest phase, with content continuing to worsen as the disease progresses. Based on previous studies, we extracted a few basic and commonly used linguistic features which are described in the following.

- Lexical features

To evaluate the lexical characteristics of the subjects’ response, we extracted the ratio of different part-of-speech (POS) and several measures of lexical features. The POS included the: ratio of nouns, verbs, and pronouns, which are basic lexical measures that describe the characteristics of a text. The lexical richness measures included type-token ratio of words and characters, which reflects word production of the subjects and has been shown to be associated with cognitive decline. These features were extracted using Stanford CoreNLP (55), a Java-based toolkit developed by the Stanford NLP Group that enables users to derive linguistic annotations. The CoreNLP currently supports word segmentation and part-of-speech tagging for eight languages including Chinese.

- Semantic features

In addition to these lexical features, we extracted two domains of semantic features that measure the speech production of the subjects. Specifically, we calculated the ratio of all the information words which include nouns, verbs, adjectives, and numerals. Moreover, we recruited volunteers to count the number of semantic information units in each transcription and used these numbers for classification. The information units are specific for the Cookie Theft picture, which are conventionally divided into four categories: people, objects, places, and actions (56). Table 5 shows the full list of these information units. The result has passed consistency check to ensure accuracy.

**Table 5:** Information units of the cookie theft.

Subjects	Boy, girl, mother
Places	Kitchen, exterior
Objects	Cupboard, cookie, curtains, jar, dishes, sink, stool, water, window, dishcloth, faucet, and floor
Actions	Boy taking or stealing a cookie Boy or stool falling Mother drying or washing dishes Water overflowing or spilling Girl asking for a cookie Mother unconcerned by or unaware of the water overflowing Children stealing cookies

### 2.3 Difference testing

To see whether the extracted acoustic or linguistic features are associated with the level of cognitive decline, we perform a permutation test on the mean of each feature. Since the study is aimed at cognitive decline detection, two difference testing tasks are performed: difference testing between HC and AD, and difference testing between HC and CI (cognitive impaired, which include both the AD and MCI subjects). The null hypothesis is that the distribution of each feature is the same for HC as for AD (or the same for HC as for CI) in our sample data. In our permutation test, we compute the observed mean value of each feature and then compare it with multiple simulated test mean values, generated through random permutations. The test mean values give a simulated distribution, so an empirical *p*-value for the observed mean value is calculated based on this simulated distribution. The mean value of each feature in HC, AD, and CI, as well as the empirical *p*-values in the two permutation tests, are listed in Table 6.

**Table 6:** Result of statistical difference analysis: the mean value of each feature in the HC, AD, CI group, and the empirical *p*-values obtained from the two permutation tests (std, standard deviation; med, median).

Feature	Mean in HC	Mean in AD	Mean in CI	Empirical *p*-value, HC vs. AD	Empirical *p*-value, HC vs. CI
F0-1	140.420	161.085	154.817	0.0205	0.0575
F0-2	149.492	173.266	163.467	0.0114	0.0829
F0-3	151.826	182.486	169.933	0.0026	0.0309
F0-4	156.101	188.991	176.081	0.0015	0.0223
F0-5	159.801	193.094	181.430	0.0026	0.0166
F0-6	163.012	200.132	186.841	0.0021	0.0182
F0-7	166.376	206.172	191.496	0.0020	0.0151
Jitter	0.038	0.045	0.041	0.0620	0.2843
Shimmer	0.167	0.175	0.171	0.2616	0.5028
MFCC1 mean	234.901	196.541	225.899	0.1144	0.7277
MFCC2 mean	24.713	61.410	31.532	0.1299	0.7726
MFCC3 mean	53.839	50.680	52.700	0.6851	0.8911
MFCC4 mean	−10.847	−1.813	−11.745	0.3319	0.9296
MFCC5 mean	−2.153	1.151	−3.947	0.5688	0.7334
MFCC6 mean	−19.508	−12.374	−16.963	0.1670	0.5954
MFCC1 std	78.217	62.003	71.342	0.0071	0.2070
MFCC2 std	49.469	41.364	46.065	0.0103	0.2946
MFCC3 std	39.740	34.426	38.318	0.0391	0.5896
MFCC4 std	33.715	29.314	31.570	0.0187	0.2230
MFCC5 std	31.452	31.229	31.718	0.8696	0.8283
MFCC6 std	27.930	26.263	27.480	0.1416	0.6374
Formant1 med	445.556	449.034	449.117	0.8454	0.8115
Formant2 med	1624.625	1711.090	1650.245	0.0120	0.3852
Formant3 med	2651.637	2782.151	2720.257	0.0024	0.0832
Formant4 med	3774.587	3859.464	3805.747	0.0416	0.4376
Ratio of nouns	0.251	0.194	0.222	0.0014	0.0804
Ratio of verbs	0.223	0.271	0.258	0.0064	0.0075
Ratio of pronouns	0.133	0.183	0.174	0.0067	0.0177
Type-token ratio of words	0.558	0.586	0.575	0.4043	0.5736
Type-token ratio of characters	0.471	0.513	0.499	0.1972	0.3483
Density of information units	0.546	0.531	0.542	0.4130	0.8255
Information units, people	2.545	2.300	2.414	0.2308	0.4791
Information units, objects	5.773	2.933	4.100	0.0000	0.0036
Information units, places	0.727	0.200	0.557	0.0006	0.2743
Information units, actions	3.136	2.133	2.700	0.0188	0.2753

The F0s and formants were measured in Hz. Other measures are dimensionless numbers or ratios.

Results of the permutation tests on acoustic features are shown in Table 6. AD and MCI patients show significant higher F0 (*p* < 0.05) compared with healthy older adults. For MFCC, only MFCC1 std., MFCC2 std., MFCC3 std., MFCC4 std., and MFCC5 std. show significant differences between AD and healthy control group. For Format, only Formant2 median, Formant3 median, and Formant4 median show significant differences between AD patients and healthy controls.

Group comparisons of linguistic features are also shown in Table 6. For linguistic feature, compared with healthy older adults, patients with AD show a significant reduction in the Ratio of nouns, Ratio of verbs and Ratio of pronouns (*p* < 0.01). There is also significant difference between control group and older adults with MCI on Ratio of verbs (*p* < 0.01) and Ratio of pronouns (*p* < 0.05). There is no significant difference between healthy controls and AD patients in Type-Token ratio of words, Type-Token ratio of characters and Density of information units (*p* > 0.05), and so does healthy controls and MCI patients. Further examination of information units reveals that AD patients show a significant reduction in references to objects, places (*p* < 0.01) and actions (*p* < 0.05). Scores for semantic units between healthy controls and MCI patients show significant difference only in objects (*p* < 0.01).

### 2.4 ML models training

After the acoustic and linguistic features were extracted from the speech data, they were used to train machine learning models. The machine learning classifiers we used include Logistic Regression (Logistic), Random Forest (RF), Support Vector Machines (SVM), Gaussian Naïve Bayesian (GNB), and k-Nearest Neighbors (kNN). The models are summarized in Table 7. For the RF, SVM, and kNN classifier, we applied grid search on the following parameters: for the RF, the number of estimators *N*; for the SVM, the kernel regularization parameter *C*; for the kNN, the number of neighbors *k*. The ML classification was implemented using Python (version 3.9.12) toolbox sklearn (57).

**Table 7:** Summary of machine learning classifiers employed in this study.

Model	Information
Logistic	L2 penalty, regularization rate = 1.0
GNB	
RF	Grid search on *N* = 3, 4, 5, …, 15
SVM	RBF kernel, *γ* = “scale,” grid search on *C* = 0.1, 0.4, 0.7, …, 2.5
kNN	Weight = “uniform,” grid search on *k* = 1, 2, 3, …, 8

We conducted two binary classification tasks: HC vs. AD, HC vs. CI. For each task we trained the machine learning models listed in Table 7 with only acoustic features, only linguistic features, and all the acoustic and linguistic features, and a combination of both acoustic and linguistic features. The purpose is to compare the efficiency of acoustic and linguistic features on cognitive decline detection.

Before the data are fed into the model, we perform z-score normalization: all the values of each feature are subtracted from their mean value and then divided by their standard deviation. We conduct normalization on the features within each gender group to reduce any potential impact of gender imbalance on classification. Normalization increases the stability of the model parameters during training and gives better accuracies.

We used Leave-One-Out method for training and validation: in each loop, one subject’s feature data was hold out, while the rest of the subjects’ feature data were used to train the machine learning model. Then, the trained model was used to predict the label of the held-out sample. The performance of the model was evaluated by the accuracy of predicting all the subjects. The sensitivity and the precision were also computed as *TP*/(*TP* + *FN*) and *TP*/(*TP* + *FP*), respectively, where *TP*, *FN*, *FP* represent the number of true positive, false negative, and false positive samples, as in a typical classification paradigm. The sensitivity was especially important to cognitive screening since it measured the models’ capabilities in detecting cognitive decline. The F1 score was also calculated based on the sensitivity and precision.

## 3 Results

In the two classification tasks, HC vs. AD classification and HC vs. CI classification, each model was trained on the acoustic feature set, the linguistic feature set, and all features combined. The accuracies are summarized in Table 8. The sensitivity and precision are summarized in Table 9.

**Table 8:** Accuracy of each machine learning model in each classification task on different feature sets (%).

HC vs. AD			
ML model	Feature set		
	Acoustic	Linguistic	All
Logistic	67.31	75.00	75.00
GNB	**73.08**	73.08	73.08
RF	67.31	75.00	75.00
SVM	69.23	**76.92**	**80.77**
kNN	67.31	75.00	76.92

HC vs. CI			
ML model	Feature set		
	Acoustic	Linguistic	All
Logistic	71.74	76.09	72.83
GNB	60.87	66.30	65.22
RF	**76.09**	78.26	76.09
SVM	**76.09**	**80.43**	**77.17**
kNN	**76.09**	81.52	**77.17**

In either of the HC vs. AD task and the HC vs. CI task, each machine learning model was trained on acoustic feature set, linguistic feature set, and all features. The highest accuracy achieved in each classification task is marked in bold.

**Table 9:** Sensitivity (in %), and precision (in %), and F1 score of each machine learning model in each classification task on different feature sets.

**ML model**	HC vs. AD								
	Feature set								
	Acoustic			Linguistic			ALL		
Logistic	68.57	80.00	0.739	77.42	80.00	0.787	79.31	76.67	0.780
GNB	70.00	93.33	**0.800**	76.67	76.67	0.767	71.05	90.00	0.794
RF	67.57	83.33	0.746	77.42	80.00	0.787	74.29	86.67	0.800
SVM	68.42	86.67	0.765	78.13	83.33	**0.807**	83.33	83.33	**0.833**
kNN	64.44	96.67	0.773	79.31	76.67	0.780	73.68	93.33	0.824
HC vs. CI									
ML model	Feature set								
	Acoustic			Lingustic			All		
Logistic	78.21	87.14	0.824	79.27	92.86	0.855	72.83	80.82	0.825
GNB	74.29	74.29	0.743	80.00	74.29	0.770	65.22	78.79	0.765
RF	77.91	95.71	0.859	81.25	92.86	0.867	76.09	79.27	0.855
SVM	76.09	100.00	**0.864**	80.23	98.57	**0.885**	77.17	77.53	0.868
kNN	77.27	97.14	0.861	88.41	87.14	0.878	77.17	76.92	**0.870**

Each cell displays sensitivity first, precision next, and finally F1 score. The highest F1 score achieved in each classification task is marked in bold.

In Table 8, the highest accuracies achieved in each classification task on each feature set are highlighted in bold. It is noteworthy that the SVM model generally outperformed other models across different tasks in terms of accuracy. For instance, in the HC vs. AD task, the SVM achieved the highest accuracy of 76.92% on the linguistic feature set and the highest accuracy of 80.77% on all features combined. In the HC vs. CI task, the SVM achieved the highest accuracy of 80.43% on the linguistic features, shared the highest accuracy of 76.09% with the kNN on the acoustic features and 77.17% on all the features. For each classification task, the models that achieved the highest accuracy on each feature set are summarized in Table 10. Related hyperparameters and the corresponding confusion matrices are displayed.

**Table 10:** Summary of the model that achieves the highest accuracy in each classification task with each feature set, with any relevant hyperparameters in the brackets and the confusion matrix below.

Classification task	Feature set		
	Acoustic	Linguistic	All
HC vs. AD	GNB 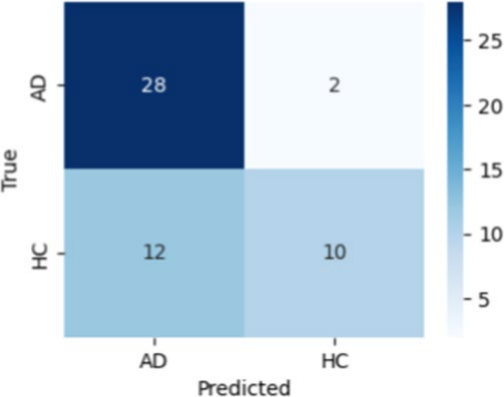	SVM (*C* = 0.1) 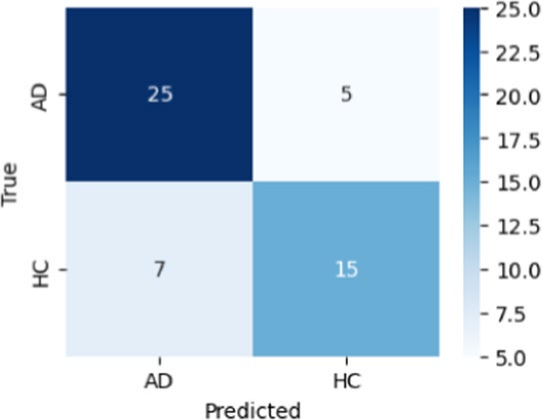	SVM (*C* = 1.3) 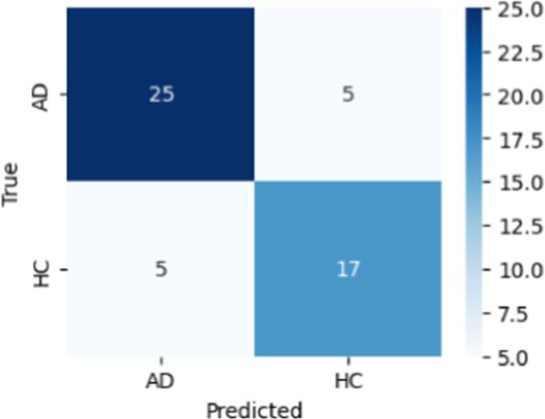
HC vs. CI	SVM (*C* = 0.1) 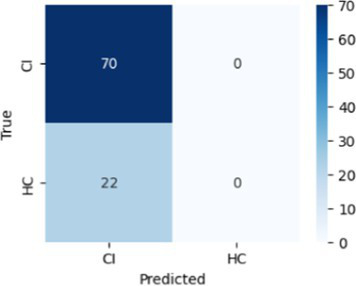	SVM (*C* = 1.9) 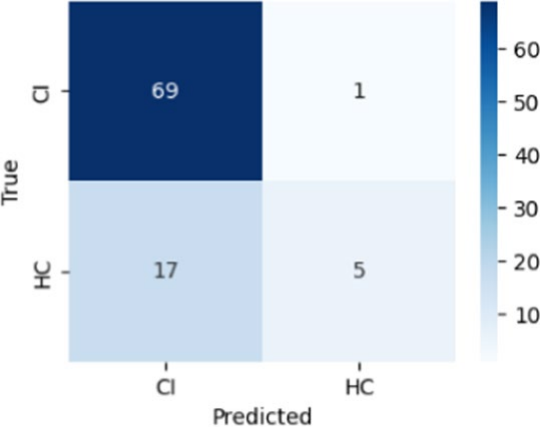	kNN (*k* = 6) 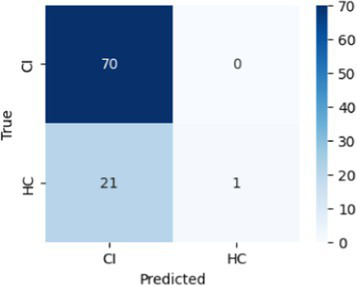

For multiple models that achieve the same highest accuracy, the one with the highest F1 score is displayed.

Additionally, the classification performance varied between different models on the same dataset. For instance, in the HC vs. AD classification, the accuracy on all the features ranged from 73.08% achieved by the GNB to 80.77% achieved by the SVM. In the HC vs. CI classification, the accuracy on all the features ranged from 65.22% achieved by the GNB to 77.17% achieved by the SVM and kNN. This variation in model performance may be attributed to several factors including characteristics of the dataset, inductive biases of the models, and hyperparameters of the models. For instance, the GNB classifier assumes independence between different features and Gaussian distributions, which may not be suitable for our feature data, since we included several similar statistics of the same acoustic feature (e.g., the seven consecutive F0s). This could possibly account for the relatively low accuracies achieved by the GNB. Yet, the SVM has been reported to perform well on small-size and high-dimensional datasets, which are exactly the characteristics of our feature data. It might be suggested that the SVM is a good candidate for cognitive screening based on multiple speech features.

In addition to the cross-model comparison, it is also intriguing to find that the models generally performed better on the linguistic feature set than on the acoustic feature set. For example, in the HC vs. AD task, the kNN achieved a remarkable accuracy of 75.00% on the linguistic features, but only 67.31% on the acoustic features; in the HC vs. CI task, an accuracy of 81.52% on the linguistic features, but only 76.09% on the acoustic features (Table 8). The possible causes for this difference in model performance on different features and its implication will be explored in section 4.

## 4 Discussion

Although a volume of research has employed acoustic and linguistic features to develop automated speech screening for AD, there seems to be a lack of comparative studies on which type of indicator is more effective, especially in Chinese context. In this study, we compared the effectiveness of acoustic features, linguistic features and their combination on automated speech screening, and developed an automated speech analysis model in Chinese context.

The accuracy achieved with linguistic features is generally higher than acoustic features and their combination for training. The highest accuracy to differentiate HC and AD is 80.77% achieved by SVM based on all the acoustic and linguistic features, while the highest accuracy to differentiate HC and AD or MCI is 80.43% achieved by SVM, using only linguistic features.

The possible reason is that the data we collected are natural speech from daily living environments but not in a studio without noise. Therefore, the values of acoustic features are influenced by background noise while the transcriptions are exact and the linguistic features are not affected. This actually matches our motivation of research. We hope to apply our research framework to cognitive impairment screening among the older adults in communities, where a relatively high ratio of noise in recordings is inevitable. From the comparison between accuracies using acoustic and linguistic features, we also suggest the linguistic feature set as a better marker in cognitive impairment screening in community settings.

Language impairment represent a prominent manifestation of AD. Numerous studies have illustrated that these subtle alternations are often quantifiable even in early phases of the disease (58, 59). Efforts have been undertaken to conduct both quantitative and qualitative investigations into the language capabilities of individuals with AD, encompassing comparative analyses between patients with AD and healthy control cohorts, as well as comparisons among patients at various disease stages. These analyses include a broad spectrum of discourse features across multiple language domains, including semantics, syntax, pragmatics and phonology (60–63).

Through the entire course of the disease, language impairment appears to occur disproportionately, with previous research indicating that deficits in semantics and pragmatics are more prevalent and pronounced compared to syntactic impairments (63). In the stage of MCI, characteristic linguistic changes include longer hesitations, decreased speech rate, and more frequent difficulties in word retrieval during spontaneous speech (27). During the mild AD stage, patients tend to speak more slowly, exhibit longer pauses, and experience increased difficulty in finding the correct words, resulting in speech disfluency or interrupted messages (64). Individuals with AD generally produce shorter texts than those in an MCI group, with less coherent information, more incoherent phrases, and increased occurrence of semantic and graphemic paraphasia (65). In the advanced stages of the disease, the syntactic structures of speech are susceptible to impairment. Disease progression often entails a simplification of syntactic complexity, characterized by the frequent utilization of simple sentences to convey fundamental semantic content. This phenomenon culminates in the production of short, repetitive, and fragmented utterances, potentially culminating in mutism (66). The disproportionate impairments in language functions observed throughout the progression of AD have been acknowledged as having significant clinical implications.

Neuro-psychological assessment tools, such as the Mini-Mental State Examination (MMSE) and the Montreal Cognitive Assessment (MoCA), exhibit high sensitivity and specificity in detecting AD. However, low scores on standard language tests cannot fully reflect actual performance of patients in daily conversations (67), and the application is often limited clinically difficulty in recognizing early symptoms and the clinicians’ insufficient time to assess cognitive impairment (68, 69). Connected speech requires diverse and complicated cognitive functions, making it sensitive to cognitive decline (70). Incorporating AI-based AD and MCI screening algorithms clinically can streamline MCI and AD diagnosis, making the screening and early detection of cognitive decline more efficient and effective, and consequently reduce the overall healthcare utilization and costs.

This theoretical study may provide the possibility of automatic speech analysis and cognitive impairment risk assessment in real-scene application, such as in community screening and on a mobile App. As far as we know, there is few such application in China currently and relevant theoretical study on Chinese speech data is insufficient. Therefore, we believe our project is a pioneering study in this field and would pave the way for further development.

While this study has preliminarily demonstrated the effectiveness of applying machine learning techniques to Chinese AD detection, there are still some limitations. Firstly, the dataset is relatively small from a computational standpoint, which restricts both the ability to train the model with extensive data and the utilization of state-of-the-art deep learning techniques. Despite validating the results through cross-validation, this constraint still imposes limitations on the classifier’s performance.

A second drawback is the limited range of features used in this study. While the 25 acoustic features and 10 linguistic features were shown to produce remarkable classification performance both respectively and altogether, this total number of features is far less than that compared with some cutting-edge research which utilizes tens and even hundreds of features. This may impede the machine learning classifiers from detecting cognitive decline from a more comprehensive perspective. Part of the reason stems from the insufficient development of Chinese algorithms at various linguistic levels. The results may vary when different acoustic and linguistic features are selected, but as far as the results of this study are concerned, the linguistic features play an important role in automated speech screening. It is expected that scholars can use different databases and richer features to validate the results of the study.

Thirdly, as an attempt at developing a Chinese language analysis model, the existing metrics fail to cover all languages levels and linguistic abilities. In the future, we aim to incorporate a broader range of linguistic features across multiple levels and collect more diverse range of language data, particularly data from different pathological stages. Leveraging an augmented dataset, the aim is to advance the precision of binary classification and explore the feasibility of ternary classification.

## 5 Conclusion

Alzheimer’s disease is one of the most challenging health problems scientists are facing since decades. In this study, we compared which kind of feature set (acoustic set, linguistic set, and their combination) has the best effectiveness on automatic speech screening and developed an automatic speech screening model for Chinese corpus. We extracted acoustic features (pitches, jitter, shimmer, MFCCs, and formants) and linguistic features (part-of-speech, type-token ratio, information words, information units) to train 5 Machine Learning algorithms including Logistic Regression, GNB, RF, SVM, and kNN to automatically detect MCI and AD. We found the accuracy with linguistic features is generally higher than acoustic features and their combination in training, demonstrating the importance of linguistic features in automated speech analysis, especially for date collected in natural living environment. This study also illustrates the applicability of automated speech screening in Chinese. The extensive experimental results show that SVM model achieved better performance in differentiating HC and AD (80.77%) as compared to other ML algorithms, and the highest accuracy to differentiate HC and AD or MCI is 80.43% achieved by RF, based only on linguistic features.

## Data availability statement

The raw data supporting the conclusions of this article will be made available by the authors, without undue reservation.

## Ethics statement

The studies involving humans were approved by the Research Ethics Committee, School of Foreign Studies, Tongji University, under the approval number tjsflrec202306. The studies were conducted in accordance with the local legislation and institutional requirements. The participants provided their written informed consent to participate in this study.

## Author contributions

LH: Conceptualization, Funding acquisition, Resources, Writing – review & editing. HY: Methodology, Writing – original draft. YC: Data curation, Writing – original draft. JY: Data curation, Writing – review & editing.

## References

[1] Long S Benoist C Weidner W. World Alzheimer report 2023: reducing dementia risk: never too early, never too late. London: Alzheimer’s Disease International (2023).

[2] Garcia SF Ritchie C Luz S. Artificial intelligence, speech and language processing approaches to monitoring Alzheimer’s disease: a systematic review. J Alzheimers Dis. (2020) 78:1547–74. doi: 10.3233/JAD-200888 33185605 PMC7836050

[3] Yamada Y Shinkawa K Nemoto M Nemoto K Arai T. A mobile application using automatic speech analysis for classifying Alzheimer’s disease and mild cognitive impairment. Comput Speech Lang. (2023) 81:101514. doi: 10.1016/j.csl.2023.101514

[4] Zolnoori M Zolnour A Topaz M. ADscreen: a speech processing-based screening system for automatic identification of patients with Alzheimer’s disease and related dementia. Artif Intell Med. (2023) 143:102624. doi: 10.1016/j.artmed.2023.102624, PMID: 37673583 PMC10483114

[5] Balagopalan A Eyre B Rudzicz F Novikova J. To BERT or not to BERT: comparing speech and language-based approaches for Alzheimer's disease detection. (2020) 2167–2171. doi: 10.21437/Interspeech.2020-2557

[6] Jawahar G Sagot B Seddah D. What does BERT learn about the structure of language? Proceedings of the 57th Annual Meeting of the Association for Computational Linguistics, (2019), 3651–7.

[7] Gonzalez-Moreira E Torres-Boza D Kairuz H Ferrer C Garcia-Zamora M Espinoza-Cuadros F. Automatic prosodic analysis to identify mild dementia. Biomed Res Int. (2015) 2015:1–6. doi: 10.1155/2015/916356, PMID: 26558287 PMC4629008

[8] Roshanzamir A Aghajan H Soleymani Baghshah M. Transformer-based deep neural network language models for Alzheimer’s disease risk assessment from targeted speech. BMC Med Inform Decis Mak. (2021) 21:92. doi: 10.1186/s12911-021-01456-3, PMID: 33750385 PMC7971114

[9] Gonzalez-Moreira E Torres-Boza D Garcia-Zamora MA Ferrer CA Hernandez-Gomez LA. Prosodic speech analysis to identify mild cognitive impairment In: VI Latin American Congress on Biomedical Engineering CLAIB 2014, Paraná, Argentina 29, 30 and 31 October 2014. Cham: Springer International Publishing (2015). 580–3.

[10] König A Satt A Sorin A Hoory R Toledo-Ronen O Derreumaux A. Automatic speech analysis for the assessment of patients with predementia and Alzheimer’s disease. Alzheimer’s & Dementia: Diagnosis, Assessment & Disease Monitoring. (2015) 1:112–124. doi: 10.1016/j.dadm.2014.11.012, PMID: 27239498 PMC4876915

[11] Hoffmann I Nemeth D Dye CD Pákáski M Irinyi T Kálmán J. Temporal parameters of spontaneous speech in Alzheimer’s disease. Int J Speech Lang Pathol. (2010) 12:29–34. doi: 10.3109/17549500903137256, PMID: 20380247

[12] Gosztolya G Vincze V Tóth L Pákáski M Kálmán J Hoffmann I. Identifying mild cognitive impairment and mild Alzheimer’s disease based on spontaneous speech using ASR and linguistic features. Comput Speech Lang. (2019) 53:181–97. doi: 10.1016/j.csl.2018.07.007

[13] Martínez-Sánchez F Meilán JJG García-Sevilla J Carro J Arana JM. Oral reading fluency analysis in patients with Alzheimer disease and asymptomatic control subjects. Neurologia. (2013) 28:325–31. doi: 10.1016/j.nrl.2012.07.012, PMID: 23046975

[14] Shah MA Szurley J Mueller M Mouchtaris T Droppo J Evaluating the vulnerability of end-to-end automatic speech recognition models to membership inference attacks. in Interspeech (2021), 891–895. doi: 10.21437/Interspeech.2021-1188

[15] Martinc M Pollak S. Tackling the ADReSS challenge: a multimodal approach to the automated recognition of Alzheimer’s dementia In: Interspeech (2020). 2157–61. doi: 10.21437/Interspeech.2020-2202

[16] Meghanani A Anoop CS Ramakrishnan AG. An exploration of log-mel spectrogram and MFCC features for Alzheimer’s dementia recognition from spontaneous speech. 2021 IEEE spoken language technology workshop (SLT), 670–7. New York, NY: IEEE (2021).

[17] Panyavaraporn J Horkaew P. Classification of Alzheimer’s disease in PET scans using MFCC and SVM. Int J Adv Sci Eng Inf Technol. (2018) 8:1829–35. doi: 10.18517/ijaseit.8.5.6503

[18] Dessouky MM Elrashidy MA Taha TE Abdelkader HM. Computer-aided diagnosis system for Alzheimer’s disease using different discrete transform techniques. Am J Alzheimers Dis Other Dement. (2016) 31:282–93. doi: 10.1177/1533317515603957 PMC10852668 26371347

[19] Abdallah-Qasaimeh B Ratté S. Detecting depression in Alzheimer’s disease and MCI by speech analysis. J Theor Appl Inf Technol. (2021) 99:1162–71.

[20] Triapthi A Chakraborty R Kopparapu SK Dementia classification using acoustic descriptors derived from subsampled signals. In 2020 28th European signal processing conference (EUSIPCO), 91–95. New York, NY: IEEE (2021).

[21] Balagopalan A Eyre B Robin J Rudzicz F Novikova J. Comparing pre-trained and feature-based models for prediction of Alzheimer’s disease based on speech. Front Aging Neurosci. (2021) 13:635945. doi: 10.3389/fnagi.2021.635945, PMID: 33986655 PMC8110916

[22] Lopez-de-Ipiña K Alonso JB Solé-Casals J Barroso N Henriquez P Faundez-Zanuy M. On automatic diagnosis of Alzheimer’s disease based on spontaneous speech analysis and emotional temperature. Cogn Comput. (2015) 7:44–55. doi: 10.1007/s12559-013-9229-9

[23] Yoshii K Nishimura M Kimura D Kosugi A Shinkawa K. A study for detecting mild cognitive impairment by analyzing conversations with humanoid robots. In 2021 IEEE 3rd global conference on life sciences and technologies (LifeTech), 347–350. New York, NY: IEEE (2021).

[24] Meilán JJG Martínez-Sánchez F Carro J López DE Millian-Morell L Arana JM. Speech in Alzheimer’s disease: can temporal and acoustic parameters discriminate dementia? Dement Geriatr Cogn Disord. (2014) 37:327–34. doi: 10.1159/000356726, PMID: 24481220

[25] Orimaye SO Wong JSM Golden KJ Learning predictive linguistic features for Alzheimer’s disease and related dementias using verbal utterances. Proceedings of the Workshop on Computational Linguistics and Clinical Psychology: From Linguistic Signal to Clinical Reality Baltimore, Maryland, USA: Association for Computational Linguistics. (2014), 78–87.

[26] Ammar RB Ayed YB. Language-related features for early detection of Alzheimer disease. Procedia Comput Sci. (2020) 176:763–70. doi: 10.1016/j.procs.2020.09.071

[27] Satt A Hoory R Konig A Aalten P Robert PH Speech-based automatic and robust detection of very early dementia. Proceedings of the Annual Conference of the International Speech Communication Association (2014), 2538–2542.

[28] Rohanian M Hough J Purver M. Multi-modal fusion with gating using audio, lexical and disfluency features for Alzheimer’s dementia recognition from spontaneous speech Proc. Interspeech, (2020), 2187–2191. doi: 10.21437/Interspeech.2020-2721

[29] Eyben F Weninger F Gross F Schuller B. Recent developments in opensmile, the Munich open-source multimedia feature extractor. In Proceedings of the 21st ACM International Conference on Multimedia (2013), 835–8.

[30] Eyben F Scherer KR Schuller BW Sundberg J André E Busso C. The Geneva minimalistic acoustic parameter set (GeMAPS) for voice research and affective computing. IEEE Trans Affect Comput. (2015) 7:190–202. doi: 10.1109/TAFFC.2015.2457417

[31] Luz S Haider F Fuente S Fromm D MacWhinney B. Alzheimer’s dementia recognition through spontaneous speech: the ADReSS challenge. Front Comput Sci. (2020) 3:780169. doi: 10.3389/fcomp.2021.780169 PMC8920352 35291512

[32] Chen J Wang Y Wang D. A feature study for classification-based speech separation at low signal-to-noise ratios. IEEE/ACM Trans Audio Speech Lang Proc. (2014) 22:1993–2002. doi: 10.1109/TASLP.2014.2359159

[33] He R Chapin K Al-Tamimi J Bel N Marquié M Rosende-Roca M. Automated classification of cognitive decline and probable Alzheimer’s dementia across multiple speech and language domains. Am J Speech Lang Pathol. (2023) 32:2075–86. doi: 10.1044/2023_AJSLP-22-00403, PMID: 37486774

[34] Szatloczki G Hoffmann I Vincze V Kalman J Pakaski M. Speaking in Alzheimer’s disease, is that an early sign? Importance of changes in language abilities in Alzheimer’s disease. Front Aging Neurosci. (2015) 7:195. doi: 10.3389/fnagi.2015.00195 26539107 PMC4611852

[35] Klumpp P Fritsch J Nöth E ANN-based Alzheimer’s disease classification from bag of words. In Speech communication; 13th ITG-symposium, 1–4. Frankfurt am Main: VDE (2018).

[36] Asgari M Kaye J Dodge H. Predicting mild cognitive impairment from spontaneous spoken utterances. Alzheimer’s Dementia Transl Res Clin Interv. (2017) 3:219–28. doi: 10.1016/j.trci.2017.01.006, PMID: 29067328 PMC5651423

[37] Jarrold W Peintner B Wilkins D Vergryi D Richey C. Aided diagnosis of dementia type through computer-based analysis of spontaneous speech. In Proceedings of the workshop on computational linguistics and clinical psychology: from linguistic signal to clinical reality. Baltimore, Maryland, USA: Association for Computational Linguistics(2014), 27–37. doi: 10.3115/v1/W14-3204

[38] Fraser KC Meltzer JA Rudzicz F. Linguistic features identify Alzheimer’s disease in narrative speech. J Alzheimers Dis. (2016) 49:407–22. doi: 10.3233/JAD-150520 26484921

[39] Kothari M Shah DV Moulya T Rao SP Jayashree R. Measures of lexical diversity and detection of Alzheimer’s using speech. ICAART. (2023) 3:806–12. doi: 10.5220/0011779000003393

[40] Bucks RS Singh S Cuerden JM Wilcock GK. Analysis of spontaneous, conversational speech in dementia of Alzheimer type: evaluation of an objective technique for analysing lexical performance. Aphasiology. (2000) 14:71–91. doi: 10.1080/026870300401603

[41] Thomas MS Dockrell JE Messer D Parmigiani C Ansari D Karmiloff-Smith A. Speeded naming, frequency and the development of the lexicon in Williams syndrome. Lang Cognit Proc. (2006) 21:721–59. doi: 10.1080/01690960500258528

[42] Guinn C Singer B Habashi A. A comparison of syntax, semantics, and pragmatics in spoken language among residents with Alzheimer’s disease in managed-care facilities In: IEEE Symposium on Computational Intelligence in Healthcare and e-health (CICARE), Orlando, FL, USA, (2014): 98–103. doi: 10.1109/CICARE.2014.7007840

[43] Sirts K Piguet O Johnson M Idea density for predicting Alzheimer’s disease from transcribed speech. In Proceedings of the 21st Conference on Computational Natural Language Learning (CoNLL 2017), Vancouve (2017). 322–332.

[44] Sadeghian R Schaffer JD Zahorian SA. Speech processing approach for diagnosing dementia in an early stage. Proc Interspeech. (2017) 2017:2705–9. doi: 10.21437/Interspeech.2017-1712

[45] Guo Z Ling Z Li Y. Detecting Alzheimer’s disease from continuous speech using language models. J Alzheimers Dis. (2019) 70:1163–74. doi: 10.3233/JAD-190452, PMID: 31322577

[46] Chien YW Hong SY Cheah WT Fu LC Chang YL An assessment system for Alzheimer's disease based on speech using a novel feature sequence design and recurrent neural network. In 2018 IEEE International Conference on Systems, Man, and Cybernetics (SMC), 3289–94. New York, NY: IEEE (2018).

[47] Fraser KC Meltzer JA Graham NL Leonard C Hirst G Black SE. Automated classification of primary progressive aphasia subtypes from narrative speech transcripts. Cortex. (2014) 55:43–60. doi: 10.1016/j.cortex.2012.12.006, PMID: 23332818

[48] Kong W. Exploring neural models for predicting dementia from language (Doctoral dissertation. Vancouver, BC: University of British Columbia (2019).

[49] Goodglass H Kaplan E Weintraub S. BDAE: the Boston diagnostic aphasia examination. Philadelphia, PA: Lippincott Williams and Wilkins (2001).

[50] ByteDance Feishu (version 7.6). (2023) Available at: https://www.feishu.cn/product/minutes

[51] Jadoul Y Thompson B Boer B. Introducing Parselmouth: a Python interface to Praat. J Phon. (2018) 71:1–15. doi: 10.1016/j.wocn.2018.07.001

[52] Vincze V Gosztolya G Tóth L Hoffmann I Detecting mild cognitive impairment by exploiting linguistic information from transcripts. Proceedings of the 54th Annual Meeting of the Association for Computational Linguistics 2 (2016) 181–187. doi: 10.18653/v1/P16-2030

[53] Ahmed S Haigh AMF de Jager CA Garrard P. Connected speech as a marker of disease progression in autopsy-proven Alzheimer’s disease. Brain. (2013) 136:3727–37. doi: 10.1093/brain/awt269, PMID: 24142144 PMC3859216

[54] Lira JOD Minett TSC Bertolucci PHF Ortiz KZ. Analysis of word number and content in discourse of patients with mild to moderate Alzheimer's disease. Dementia Neuropsychol. (2014) 8:260–5. doi: 10.1590/S1980-57642014DN83000010, PMID: 29213912 PMC5619403

[55] Manning CD Surdeanu M Bauer J Finkel J Bethard SJ McClosky D. The Stanford CoreNLP natural language processing toolkit. In Proceedings of the 52nd Annual Meeting of the Association for Computational Linguistics: System Demonstrations (2014), 55–60. doi: 10.3115/v1/P14-5010

[56] Croisile B Ska B Brabant MJ Duchene A Lepage Y Aimard G. Comparative study of oral and written picture description in patients with Alzheimer's disease. Brain Lang. (1996) 53:1–19. doi: 10.1006/brln.1996.0033, PMID: 8722896

[57] Pedregosa F Eickenberg M Ciuciu P Thirion B Gramfort A. Data-driven HRF estimation for encoding and decoding models. NeuroImage. (2015) 104:209–20. doi: 10.1016/j.neuroimage.2014.09.060, PMID: 25304775

[58] Hier DB Hagenlocker K Shindler AG. Language disintegration in dementia: effects of etiology and severity. Brain Lang. (1985) 25:117–33. doi: 10.1016/0093-934X(85)90124-5, PMID: 2411334

[59] Lihe H Che Y. Pragmatic impairment and multimodal compensation in older adults with dementia. Language and Health 1.1. (2023) 176:44–57. doi: 10.1016/j.laheal.2023.06.004

[60] Juncos-Rabadán O Facal D Rodríguez MS Pereiro AX. Lexical knowledge and lexical retrieval in ageing: insights from a tip-of-the-tongue (TOT) study. Lang Cognit Proc. (2010) 25:1301–34. doi: 10.1080/01690961003589484

[61] Karalı FS Maviş İ Cinar N. Comparison of language and narrative features of individuals among amnestic mild cognitive impairment and healthy adults. Curr Psychol. (2023) 42:25584–93. doi: 10.1007/s12144-022-03669-9

[62] Sajjadi SA Patterson K TomekM NPJ. Abnormalities of connected speech in semantic dementia vs. Alzheimer’s disease. Aphasiology. (2012) 26:847–66. doi: 10.1080/02687038.2012.654933

[63] Bayles KA Boone DR. The potential of language tasks for identifying senile dementia. J Speech Hear Disord. (1982) 47:210–7. doi: 10.1044/jshd.4702.210 7176601

[64] Huang L Yang J. An Analysis of Oral Non-Fluency of Chinese DAT Patients. Contemporary Linguistics. (2022)192–207.

[65] Taler V Phillips NA. Language performance in Alzheimer’s disease and mild cognitive impairment: a comparative review. J Clin Exp Neuropsychol. (2008) 30:501–56. doi: 10.1080/13803390701550128 18569251

[66] Hamilton HE. Conversations with an Alzheimer’s patient: an interactional sociolinguistic study. Cambridge: Cambridge University Press (1994).

[67] Slegers A Filiou RP Montembeault M Brambati SM. Connected speech features from picture description in Alzheimer’s disease: a systematic review. J Alzheimers Dis. (2018) 65:519–42. doi: 10.3233/JAD-170881, PMID: 30103314

[68] Judge D Roberts J Khandker R Ambegaonkar B Black CM. Physician perceptions about the barriers to prompt diagnosis of mild cognitive impairment and Alzheimer’s disease. Int J Alzheimers Dis. (2019) 2019:1–6. doi: 10.1155/2019/3637954 PMC6556253 31263595

[69] Lion KM Szcześniak D Bulińska K Evans SB Evans SC Saibene FL. Do people with dementia and mild cognitive impairments experience stigma? A cross-cultural investigation between Italy, Poland and the UK. Aging Ment Health. (2020) 24:947–55. doi: 10.1080/13607863.2019.1577799, PMID: 30789028

[70] Mueller KD Koscik RL Hermann BP Johnson SC Turkstra LS. Declines in connected language are associated with very early mild cognitive impairment: results from the Wisconsin registry for Alzheimer’s prevention. Front Aging Neurosci. (2018) 9:437. doi: 10.3389/fnagi.2017.00437, PMID: 29375365 PMC5767238

