# Supplementary material for: Automatic speech analysis for detecting cognitive decline of older adults
Source: Front Public Health. 2024 Aug 8;12:1417966. doi: 10.3389/fpubh.2024.1417966 (PMC11338907; doi:10.3389/fpubh.2024.1417966)
Supplement: Supplementary file 1 [file Data_Sheet_1.pdf]

Appendix 1. Detailed information of all subjects

subject	gender	age	education	MOCA-B	Boston-Naming	Verbal Fluency
AD1	female	86	9	10	7	7
AD2	female	82	0	6	10	2
AD3	female	63	9	5	11	3
AD4	female	85	0	7	11	10
AD5	female	83	9	5	11	6
AD6	female	74	5	10	13	10
AD7	female	77	16	11	14	7
AD8	female	75	9	9	14	17
AD9	female	54	9	14	15	10
AD10	male	55	12	12	16	6
AD11	female	62	3	12	16	11
AD12	female	55	9	12	17	8
AD13	female	79	16	16	17	8
AD14	female	62	8	11	18	7
AD15	female	74	12	11	18	4
AD16	male	81	0	12	19	8
AD17	female	75	0	7	19	3
AD18	male	71	12	9	20	4
AD19	male	77	9	15	20	14
AD20	female	78	9	12	20	10
AD21	female	71	9	7	20	6
AD22	male	82	16	14	21	8
AD23	male	77	16	9	21	8
AD24	male	78	6	11	22	11
AD25	male	84	16	5	22	7
AD26	male	78	12	8	23	8
AD27	male	62	12	11	24	9
AD28	male	80	12	13	/	/
AD29	female	65	3	13	/	/
AD30	female	74	5	4	/	/
HC1	female	53	12	23	18	16
HC2	male	53	5	20	20	15
HC3	female	61	0	21	20	13
HC4	female	62	12	25	20	9
HC5	male	69	6	20	21	11
HC6	female	65	9	27	21	17
HC7	male	53	16	27	23	20
HC8	female	55	16	27	23	15
HC9	female	61	9	23	24	21
HC10	female	68	9	23	24	14

HC11	male	64	9	26	25	16
HC12	male	70	9	25	25	11
HC13	male	65	14	26	26	12
HC14	male	68	12	25	26	15
HC15	female	71	16	25	27	13
HC16	male	69	16	27	28	12
HC17	male	67	16	28	/	/
HC18	male	64	10	25	/	/
HC19	male	66	15	26	/	/
HC20	male	73	3	25	/	/
HC21	female	68	12	26	/	/
HC22	female	84	12	24	/	/
MCI1	female	63	0	15	13	12
MCI2	female	74	12	19	15	16
MCI3	male	65	9	17	16	13
MCI4	female	53	9	21	16	8
MCI5	female	78	9	16	19	9
MCI6	male	87	9	16	20	8
MCI7	female	74	0	17	20	18
MCI8	female	66	9	21	20	15
MCI9	male	75	9	20	21	13
MCI10	female	55	15	23	21	15
MCI11	female	63	12	22	21	17
MCI12	male	75	13	17	22	5
MCI13	male	62	12	19	23	11
MCI14	male	55	9	21	23	6
MCI15	male	55	15	17	23	9
MCI16	male	78	9	21	24	15
MCI17	female	77	10	17	24	14
MCI18	female	76	15	24	24	23
MCI19	male	80	9	21	25	13
MCI20	male	60	16	23	26	19
MCI21	male	83	16	21	26	14
MCI22	female	72	9	19	26	14
MCI23	female	63	9	22	26	14
MCI24	female	65	12	21	26	14
MCI25	female	79	16	23	26	12
MCI26	female	73	9	22	26	19
MCI27	male	68	9	20	27	10
MCI28	male	67	16	19	28	12
MCI29	female	79	16	23	28	14
MCI30	male	72	15	24	30	17
MCI31	female	67	0	16	/	/
MCI32	male	73	12	22	/	/

MCI33	male	71	9	20	/	/
MCI34	male	75	15	23	/	/
MCI35	female	74	12	16	/	/
MCI36	female	65	14	23	/	/
MCI37	female	77	9	16	/	/
MCI38	female	66	9	20	/	/
MCI39	female	70	12	20	/	/
MCI40	female	66	9	22	/	/
